# Evolution of Characteristic Aroma During Processing of Space-Induced Mutant Dahongpao Tea and the Preliminary Exploration of Its Olfactory Perception

**DOI:** 10.3390/foods15162816

**Published:** 2026-08-12

**Authors:** Junbin Gu, Shunxian Lin, Yuhua Wang, Yulin Wang, Miao Jia, Qi Zhang, Xiaoli Jia, Tingting Wang, Jianghua Ye, Haibin Wang

**Affiliations:** 1 College of Life Science, Longyan University, Longyan 364012, China; gujunbin020@outlook.com (J.G.); lsx1215@outlook.com (S.L.); yulinwang2006@outlook.com (Y.W.); wangtingting0427@outlook.com (T.W.); 2 College of Food Engineering, Zhangzhou Institute of Technology, Zhangzhou 363000, China; 3 College of Life Science, Fujian Agriculture and Forestry University, Fuzhou 350002, China; 22427025012@fafu.edu.cn (Y.W.); jiam202309@outlook.com (M.J.); 4 Fujian Key Laboratory of Big Data Application and Intellectualization for Tea Industry, College of Tea and Food Science, Wuyi University, Wuyishan 354300, China; zhangqi1113@wuyiu.edu.cn (Q.Z.); jiaxl2010@wuyiu.edu.cn (X.J.)

**Keywords:** space-induced mutant Dahongpao, volatile organic compounds, aroma transformation, molecular docking, olfactory receptor

## Abstract

Space-induced mutation is a novel approach in tea plant breeding, but its effect on processing-induced aroma formation remains unclear. This study analyzed the dynamic changes in VOCs during the processing of space-induced mutants of Dahongpao tea. The results showed that processing significantly reshaped the VOC profile, with the fermentation stage exerting the greatest impact and terpenoids serving as the major contributors. Seven characteristic VOCs (α-phellandrene, β-phellandrene, d-sylvestrene, iso-geraniol, seudenone, hexyl benzene, and nerol) were identified and a transformation network linking floral, green, woody, almond, and fruity attributes was constructed. Molecular docking revealed that VOCs exhibiting green, woody and almond characteristics could form strong binding interactions with more than 100 olfactory receptors, whereas those with floral and fruity characteristics bound to fewer receptors. This finding indicates that during the processing, the first three odor characteristics may be perceived more prominently. Overall, this study provided a theoretical basis for the aroma evaluation of space-induced mutant tea.

## 1. Introduction

Tea (*Camellia sinensis*) is one of the most popular beverages worldwide, and its aroma quality is a key determinant of consumer acceptance and economic value [1]. Wuyi rock tea, particularly Dahongpao, is renowned for its unique and complex aroma characteristics, which mainly originate from volatile organic compounds (VOCs) generated via a series of intricate biochemical reactions during processing [2]. Traditional tea processing involves steps such as withering, fermentation, kneading, and drying, each of which profoundly influences the composition and content of VOCs through enzymatic oxidation, thermal degradation, glycoside hydrolysis, and other pathways [3,4]. However, how to further optimize tea aroma quality through breeding strategies while maintaining traditional quality characteristics remains an important topic in tea plant research.

Space-induced mutation breeding exploits the unique physicochemical conditions of space (e.g., cosmic radiation, microgravity, and high vacuum) to induce random genomic mutations, thereby generating new germplasms with desirable traits [5]. In recent years, this technique has been successfully applied to various crops, including rice, wheat, and cotton, and has also shown promise in tea breeding [6,7]. The space-induced mutant Dahongpao was obtained by exposing its seeds to space aboard a retrievable satellite, followed by ground-based screening. Previous studies have indicated that space-induced mutation can significantly alter the secondary metabolite profiles of tea plants, particularly the levels of aroma-related terpenoids [8]. However, systematic investigations into the specific effects of space-induced mutation on the dynamic evolution of VOCs during tea processing, as well as on how it shapes the final aroma characteristics, are still lacking.

The effects of processing on tea VOCs have been widely documented. For instance, Jia et al. [9] found that terpenes and esters significantly increased during the fermentation stage of traditional Dahongpao tea. Qi et al. [10] indicated that withering and kneading promote the release of glycosidically bound aroma precursors. However, most of these studies have focused on conventional varieties, whereas space-induced mutant materials, owing to their genomic instability, may exhibit VOC response patterns markedly different from those of traditional varieties. Previous studies have systematically compared the differences in VOC profiles between mutant strains and their parent varieties at the stages of fresh leaves and finished tea, confirming the significant impact of mutagenesis on aroma composition [8]. On this basis, the present study further focuses on the dynamic evolution of VOC profiles throughout the whole processing chain, systematically comparing the VOC spectra at different processing stages. This approach helps to elucidate the regulatory mechanisms through which mutation breeding influences tea aroma quality. Identifying characteristic VOCs closely associated with tea aroma quality is fundamental to aroma research [11]. Previous studies have mainly focused on screening differential metabolites, often overlooking the dynamic changes in compounds during processing [12]. Effectively identifying groups of VOCs that exhibit similar trends during processing, and then pinpointing candidate compounds that show regular changes along the processing sequence, can enable more accurate identification if characteristic VOCs that contribute most to aroma quality. More importantly, the perception of tea aroma ultimately depends on the specific recognition between VOCs and human olfactory receptor proteins [13]. In recent years, molecular docking techniques have been widely used to predict the binding modes and affinities between small-molecule ligands and olfactory receptors [14]. By calculating binding energy and closest contact distance, the ease with which different VOCs are perceived by the olfactory system can be assessed. For example, Xiao et al. [15] used molecular docking to reveal the interaction mechanisms between aldehydes and the OR1A1 receptor; Zeng et al. [16] elucidated the molecular interaction mechanisms between caramel-like odorants and olfactory receptors, identifying key olfactory receptors OR1G1 and OR52H1. However, in the field of tea research, large-scale docking simulations between characteristic VOCs screened during processing and human olfactory receptors remain scarce. In particular, for space-induced mutant tea, how its unique aroma components interact with olfactory receptors and how such interactions account for the sensory characteristics of the final product have yet to be clarified.

Accordingly, this study utilized space-induced mutant Dahongpao as the experimental material to systematically track the dynamic changes in VOCs across five key processing stages (fresh leaves, withering, fermentation, kneading, and drying). The objectives were to elucidate the reshaping effect of the processing procedure on the VOC profile of space-induced mutant Dahongpao, to identify the critical processing stages and major compound classes, to screen for characteristic VOCs exhibiting regular variation patterns along the processing sequence, and to tentatively construct their aroma transformation network as a hypothetical model. Furthermore, through molecular docking simulation prediction tools, the binding affinity of the characteristic VOC to 428 human olfactory receptors was evaluated, and the potential olfactory perception mechanism was preliminarily explored. The findings will provide a theoretical basis for the aroma quality evaluation of space-induced mutant tea cultivars and offer scientific guidance for the targeted regulation of tea processing techniques.

## 2. Materials and Methods

### 2.1. Chemicals and Instruments

Conventional chemical reagents (e.g., NaCl) used in this study were of analytical grade and obtained from Sinopharm Group (Beijing, China). The main instruments and equipment included headspace vials (Agilent, Palo Alto, CA, USA), an automated solid-phase microextraction system (HS-SPME, SPME Arrow, CTC Analytics AG, Zwingen, Switzerland), a conditioning device (CTC Analytics AG, Zwingen, Switzerland), DVB/CWR/PDMS extraction fibers (Agilent, Palo Alto, CA, USA), DB-5MS capillary column (30 m × 0.25 mm × 0.25 μm, Agilent, CA, USA), a gas chromatograph (GC, 8890B, Agilent, CA, USA), and a mass spectrometer (MS, 7000D, Agilent, CA, USA).

### 2.2. Sample Collection

The space-induced mutant of *Camellia sinensis* (cv. Dahongpao) used in this study was cultivated at the Tea Tree Space Breeding Experimental Base of Xianming Wuyi Rock Tea Factory, Wuyishan City, Fujian Province, China (117°59′47.7″ E, 27°44′8.4″ N). Details of the space-induced mutation procedure are provided in the Supplementary Materials (Figure S1). In May 2025, fresh leaves of the space-induced mutant Dahongpao plants were harvested according to the traditional plucking standard of one bud with three leaves. Three independent biological replicates were used for each treatment, with 50 kg of fresh leaves per replicate. The fresh leaves were processed according to the primary processing method for Wuyi rock tea [17]. Tea samples were collected at five key processing stages: fresh leaves (FL), withering (WD), fermentation (FJ), kneading (RN), and drying (MC). Three independent replicates were used for each processing stage, with 1 kg of sample per replicate. The detailed processing procedure is illustrated in Figure S2. In brief, fresh tea leaves were first withered under a light intensity of 57,000 lux for 30 min, and then subjected to fermentation. The fermentation was carried out at 27 ± 1 °C and 70 ± 5% relative humidity for 600 min. After fermentation, the leaves were green-removed at 260 °C for 8 min, followed by kneading at 52 r/min for 8 min at 27 ± 1 °C, and finally dried at 110 °C for 80 min. Volatile organic compounds (VOCs) from tea samples at each processing stage were extracted using HS-SPME and then subjected to qualitative and quantitative analysis by GC-MS.

### 2.3. Collection and Identification of Tea VOCs

Tea samples from each processing stage were rapidly frozen in liquid nitrogen and ground into a homogeneous powder. An accurately weighed 500 mg aliquot of the powder was placed into a 20 mL headspace vial, and then 10 mL of saturated sodium chloride solution and 20 µL of internal standard solution (3-hexanone-2,2,4,4-d_4_, 10 μg/mL) were added. HS-SPME was carried out using a 120 μm DVB/CWR/PDMS fiber, with three independent replicates for each sample. Prior to extraction, the fiber was conditioned at 250 °C in the injection port for 2 h. During extraction, the sample vial was equilibrated in a 60 °C water bath for 5 min, after which the fiber was inserted and exposed to the headspace for 15 min. The fiber was then inserted into the injection port for GC-MS analysis.

VOCs analysis was performed using an Agilent 8890B gas chromatograph coupled with an Agilent 7000D mass spectrometer. Chromatographic separation was conducted on a DB-5MS capillary column using high-purity helium as the carrier gas at a flow rate of 1.2 mL/min. The injection port temperature was set to 250 °C, and the injection was performed in splitless mode with a desorption time of 5 min and a solvent delay of 3.5 min. The column temperature program was as follows: initial temperature of 40 °C with a hold time of 3.5 min, increased to 100 °C at 10 °C/min, then to 180 °C at 7 °C/min, and finally to 280 °C at 25 °C/min, with a final hold of 5 min. The mass spectrometer was operated in electron impact mode (EI) with an ionization energy of 70 eV. The ion source temperature was set to 230 °C, the transfer line temperature to 280 °C, and the quadrupole temperature to 150 °C. Data acquisition was performed in selected ion monitoring mode.

Qualitative analysis of VOCs was performed by matching against the NIST20 standard mass spectral library. Compounds were identified based on the presence of two to three characteristic ions after background subtraction, as well as retention times consistent with those of standard spectra [18]. To ensure data reliability, the following filtering criteria were applied: (1) retention index (RI) filtering using a series of n-alkanes (C_6_–C_40_), only hits with RI deviations within ±30 relative to the NIST library values were retained; (2) manual screening to remove common background contaminants (e.g., siloxanes from septum bleed, plasticizers, and column bleed fragments) based on blank runs and typical mass spectral features; After these steps, the remaining compounds were used for subsequent statistical evaluation. For quantitative analysis, the area normalization method was employed: semi-quantification was based on the peak areas of characteristic ions, and the relative content of each compound was calculated as the percentage of its peak area relative to the total peak area of all detected VOCs [8].

### 2.4. Molecular Docking Simulation of Characteristic VOCs with Human Olfactory Receptor Proteins

The three-dimensional structures of characteristic VOCs (ligands) were retrieved from the PubChem database in SDF format (https://pubchem.ncbi.nlm.nih.gov, accessed on 21 August 2025). Information on human olfactory receptor proteins (receptors) was retrieved from the Human Olfactory Data Explorer database (http://genome.weizmann.ac.il/horde/, accessed on 29 August 2025), comprising a total of 428 olfactory receptors [19]. The three-dimensional structures of the receptor proteins were downloaded from the AlphaFold Protein Structure Database in PDB format (https://alphafold.ebi.ac.uk/, accessed on 12 September 2025).

Geometric binding pocket prediction for the receptor proteins was performed using Fpocket software (v4.0) to automatically identify potential binding sites [20]. For each receptor, the top three scoring pockets were selected. A docking search grid was constructed based on the geometric center and volume of each pocket, with an additional extension of approximately 6 Å in each direction to fully accommodate the ligand molecule. Molecular docking calculations were performed using AutoDock Vina (v1.2.0), with input files (ligands and receptors) prepared using AutoDockTools (v1.5.7) [21]. Prior to docking, both ligands and receptors were preprocessed, including removal of water molecules, addition of hydrogen atoms, and assignment of charges. For receptor proteins, non-standard residues were removed and redundant conformations were cleaned using AutoDockTools. Docking results were visualized using PyMOL software (v2.5.7) to illustrate the interaction patterns between ligands and receptor binding sites.

### 2.5. Statistical Analysis

Raw data of tea VOCs were preprocessed using Excel 2020, and then statistical analysis and visualization were conducted using RStudio (v4.2.3) [22]. The specific analytical methods and corresponding R packages used are detailed as follows: stacked bar plot analysis for VOC classification (ggplot2 3.5.1), box plot analysis for VOC content (gghalves 0.1.4), principal component analysis (PCA) for VOC content (ggbiplot 0.55), K-means clustering analysis for differential VOC content (stats 4.2.0), orthogonal partial least squares discriminant analysis (OPLS-DA) modeling for differential VOC (ropls and mixOmics), bubble characteristic plot screening for characteristic VOC (ggplot2 3.4.0), and odor wheel plotting for characteristic VOC (circlize 0.4.15 and vegan 2.6.4). Statistical differences were assessed by ANOVA, with the significance level set at *p* < 0.05. Prior to conducting the ANOVA, the Shapiro–Wilk test was used to assess data normality, and Levene’s test was used to evaluate the homogeneity of variances ensuring compliance with the assumptions of ANOVA. For the OPLS-DA model, model performance was evaluated through 7-fold cross-validation and permutation testing (*n* = 200) to avoid overfitting; model quality was assessed using the R^2^Y and Q^2^ parameters.

## 3. Results and Discussion

### 3.1. Effects of Processing on Tea VOCs

**Figure 1:**
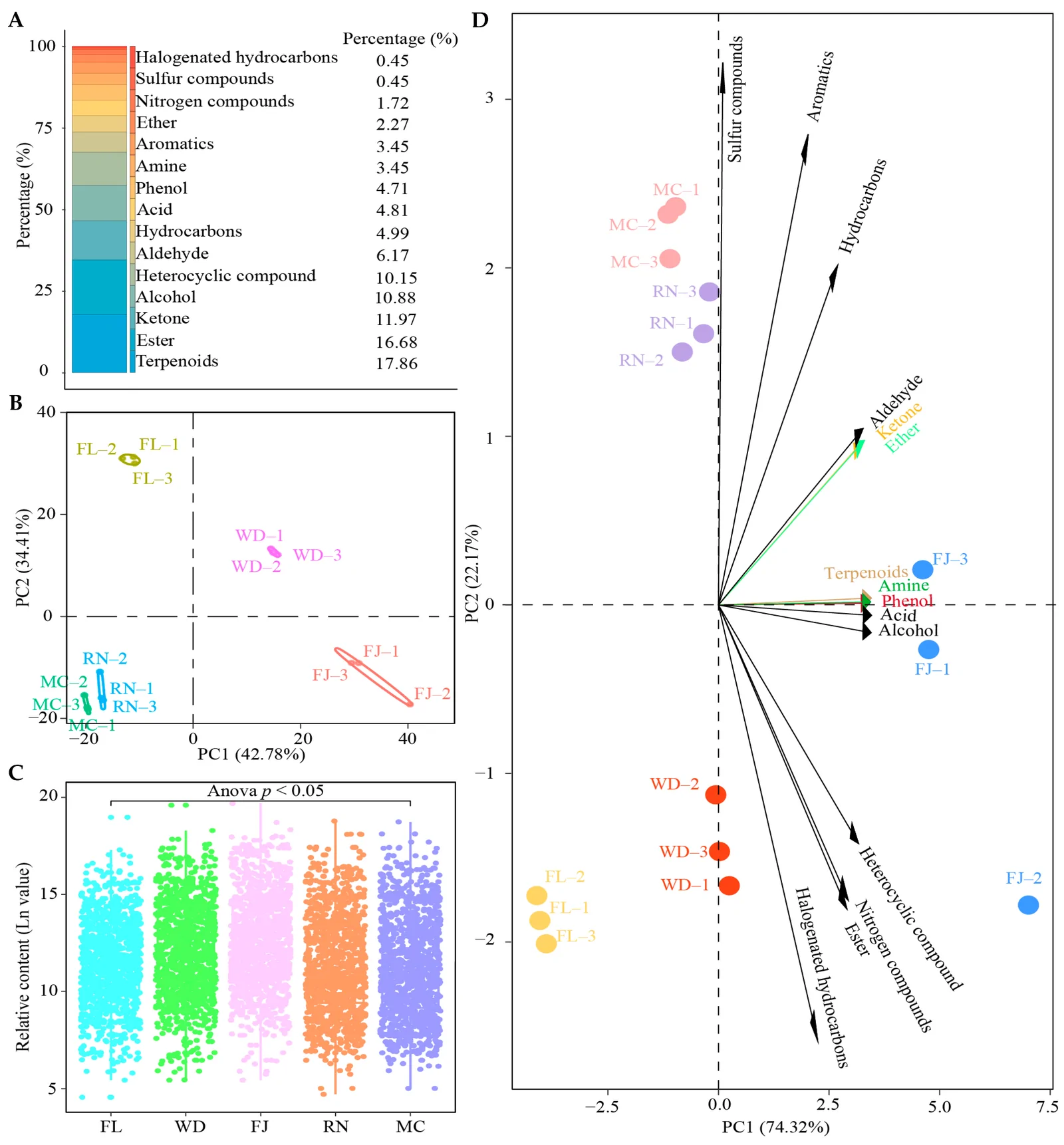
Composition and content analysis of VOCs during the processing of space-induced mutant Dahongpao tea. FL: Fresh leaves; WD: Withering; FJ: Fermentation; RN: Kneading; MC: Drying. (**A**) Composition and proportional distribution of VOCs in tea samples. (**B**) PCA of different processing stages based on VOCs profiles. (**C**) Total VOCs content at different processing stages. (**D**) PCA of classified VOCs at different processing stages.

Tea processing is a critical step in shaping the unique aroma quality of tea, as it significantly influences the composition and content of VOCs through a series of biochemical reactions, ultimately determining the aroma characteristics of the final product [23]. In this study, the dynamic changes in VOCs during the processing of space-induced mutant Dahongpao tea were systematically analyzed. The results (Figure 1A, Table S1) revealed that a total of 1103 VOCs were detected across the entire processing chain, belonging to 15 chemical classes. Among these, terpenoids, esters, and ketones were the most abundant, accounting for 17.86%, 16.68%, and 11.97% of the total, respectively. PCA revealed significant differences in VOC composition among samples from different processing stages (Figure 1B). The first two principal components accounted for 77.19% of the total variance, effectively distinguishing each processing stage, indicating that the processing procedure was the major factor driving changes in the VOC profile. Significant differences were also observed in total VOC content across processing stages (Figure 1C), which is consistent with previous findings that processing significantly alters the content and composition of tea VOCs [24].

To further investigate the variation patterns of different VOC classes across processing stages, PCA was performed on the classified VOCs (Figure 1D). The results indicated that the FL and WD stages had a greater influence on the accumulation of halogenated hydrocarbons; the FJ stage significantly affected the content and composition of terpenoids, amines, phenols, acids, and alcohols; whereas the RN and MC stages had a more pronounced impact on sulfur compounds. Fermentation, as a core step in tea processing, facilitates the transformation and accumulation of numerous aroma precursors through enzymatic oxidation reactions, profoundly altering the composition and abundance of VOCs and laying a critical foundation for the formation of the final aroma characteristics [25,26]. Overall, during the processing of space-induced mutant Dahongpao tea, the content and composition of VOCs changed significantly, with the fermentation stage exerting the most substantial influence on the overall VOC profile, thereby representing a key stage in the formation of aroma quality.

### 3.2. Screening of Characteristic VOCs During Processing

**Figure 2:**
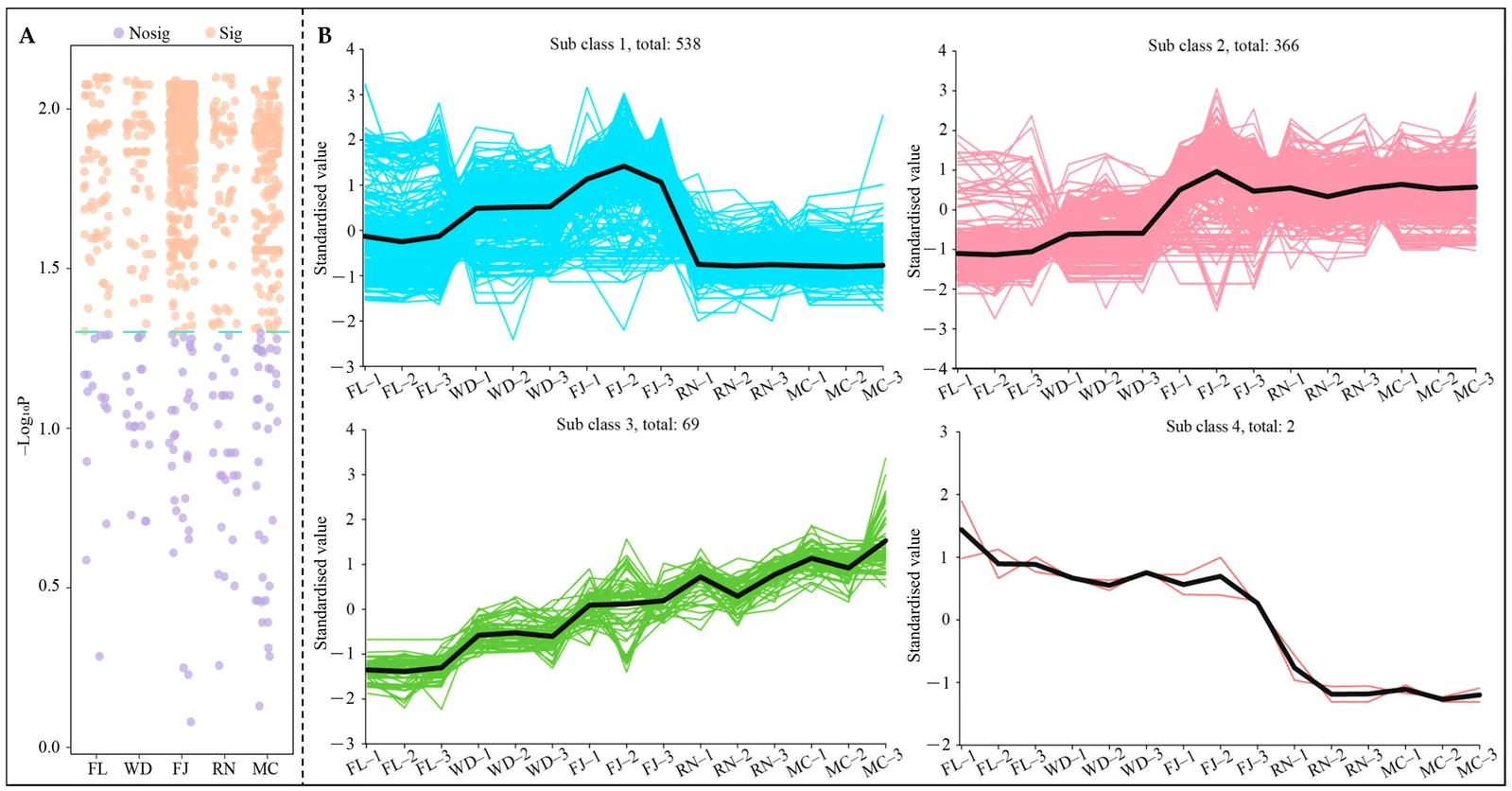
Screening of differential VOCs during the processing of space-induced mutant Dahongpao tea. FL: Fresh leaves; WD: Withering; FJ: Fermentation; RN: Kneading; MC: Drying. (**A**) Differential significance analysis of VOCs variations across different processing stages. (**B**) K-means clustering analysis of VOCs across different processing stages.

In this study, the Kruskal–Wallis test was employed to assess the significance of differences in VOC content among different processing stages. The results (Figure 2A) showed that of the total 1103 VOCs, 975 exhibited significant differences across processing stages (*p* < 0.05), while only 128 showed no significant differences, indicating that the processing procedure exerts a highly significant effect on the volatile composition of tea. Each processing step may substantially alter the aroma composition through enzymatic reactions, thermal degradation, or oxidation [27]. Further analysis of the content variations in the 975 differential VOCs using K-means clustering (Figure 2B) revealed that, as the processing progressed, 538 VOCs exhibited an initial increase followed by a decrease, 366 showed fluctuating patterns, 69 displayed a continuous increase, and 2 showed a continuous decrease. The 71 compounds that exhibited either a continuous increase or decrease were classified into 14 chemical classes, among which terpenoids were the most abundant, accounting for 27.78% (Figure S3). As important contributors to tea aroma, terpenoids typically impart floral, fruity, or woody notes to tea [12]. Their continuous changes during processing may be attributed to the combined effects of enzymatic reactions, oxidative reactions, and microbial metabolism, which promote the transformation and accumulation of volatile monoterpenes and sesquiterpenes [28]. Thus, the processing procedure significantly influences the VOC content in space-induced mutant Dahongpao tea, playing a particularly important regulatory role in the accumulation and variation of terpenoid compounds.

**Figure 3:**
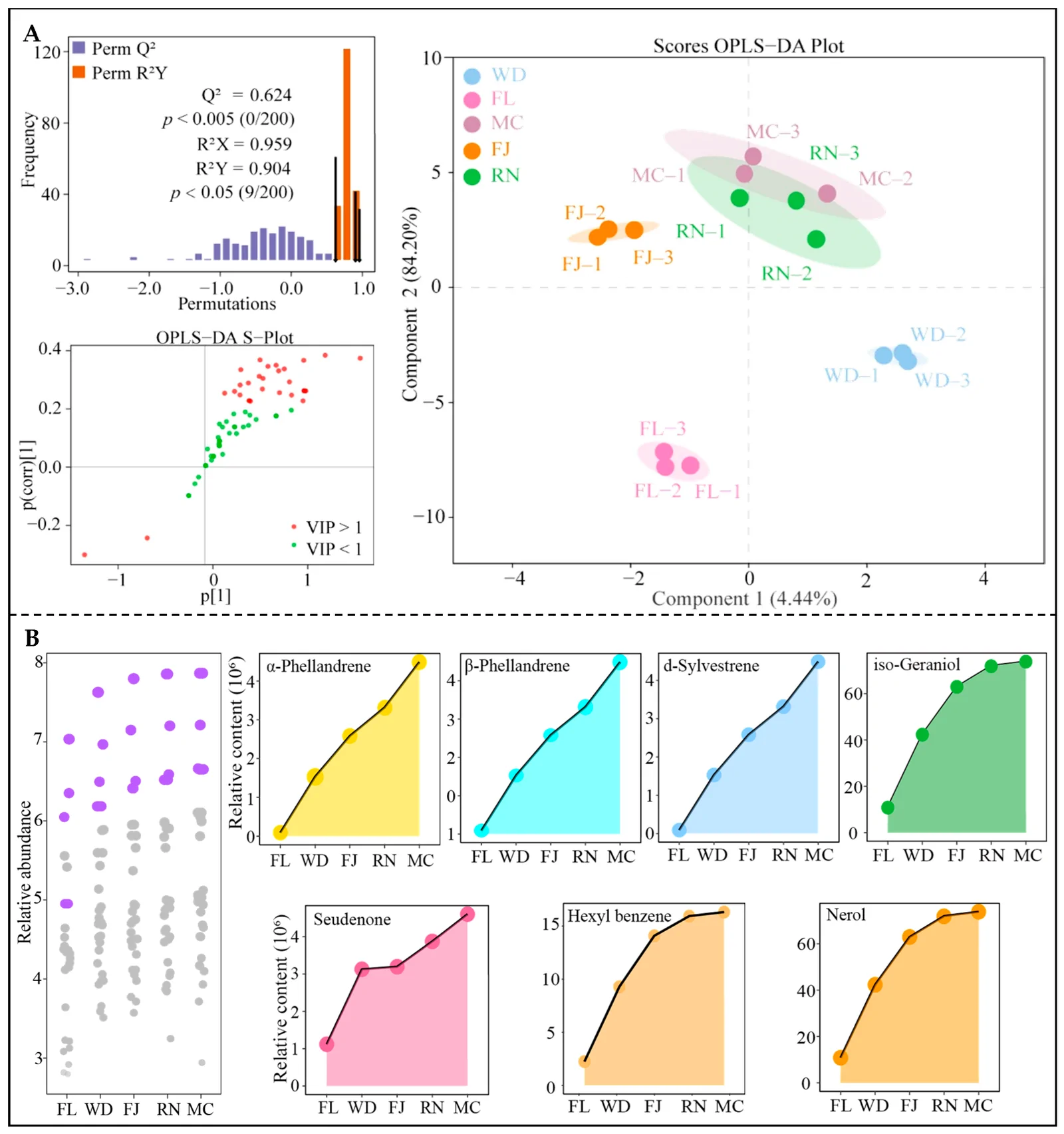
Screening and content analysis of characteristic VOCs during the processing of space-induced mutant Dahongpao tea. FL: Fresh leaves; WD: Withering; FJ: Fermentation; RN: Kneading; MC: Drying. (**A**) OPLS-DA model constructed based on VOCs content across different processing stages to screen key differential VOCs (Left: permutation test plot and S-Plot; Right: scores plot). (**B**) Bubble characteristic plot analysis of key differential VOCs to identify characteristic VOCs and analyze their contents.

On this basis, an OPLS-DA model was constructed using the 71 VOCs that exhibited significant and regular changes during processing. The model demonstrated good goodness-of-fit (R^2^Y = 0.904, *p* < 0.05) and significant predictive ability (Q^2^ = 0.624, *p* < 0.005) (Figure 3A). A total of 31 key VOCs with variable importance in projection (VIP) values greater than 1 were screened using this model, of which 29 exhibited an increasing trend during processing, while only 2 showed a decreasing trend. Further analysis of these 31 key VOCs using bubble characteristic plots identified characteristic VOCs that exhibited significant changes at different processing stages (Figure 3B), resulting in the selection of seven compounds, such as α-phellandrene, β-phellandrene, d-sylvestrene, iso-geraniol, seudenone, hexyl benzene, and nerol. These characteristic VOCs are considered to have important potential contributions to tea aroma formation. Among them, α-phellandrene and β-phellandrene exhibit green odors [29], iso-geraniol and nerol contribute floral notes [30], d-sylvestrene imparts a woody aroma [31], hexyl benzene exhibits an almond-like scent [32], and seudenone contributes fruity characteristics [33]. Meanwhile, the study revealed (Figure 3B) that the contents of the seven characteristic VOCs exhibited a significant increasing trend throughout the processing progression. In summary, during the processing of space-induced mutant Dahongpao tea, the VOCs in the leaves undergo significant changes that collectively shape its unique aroma profile, among which the seven characteristic VOCs identified above play particularly critical roles.

### 3.3. Aroma Analysis of Characteristic VOCs

**Figure 4:**
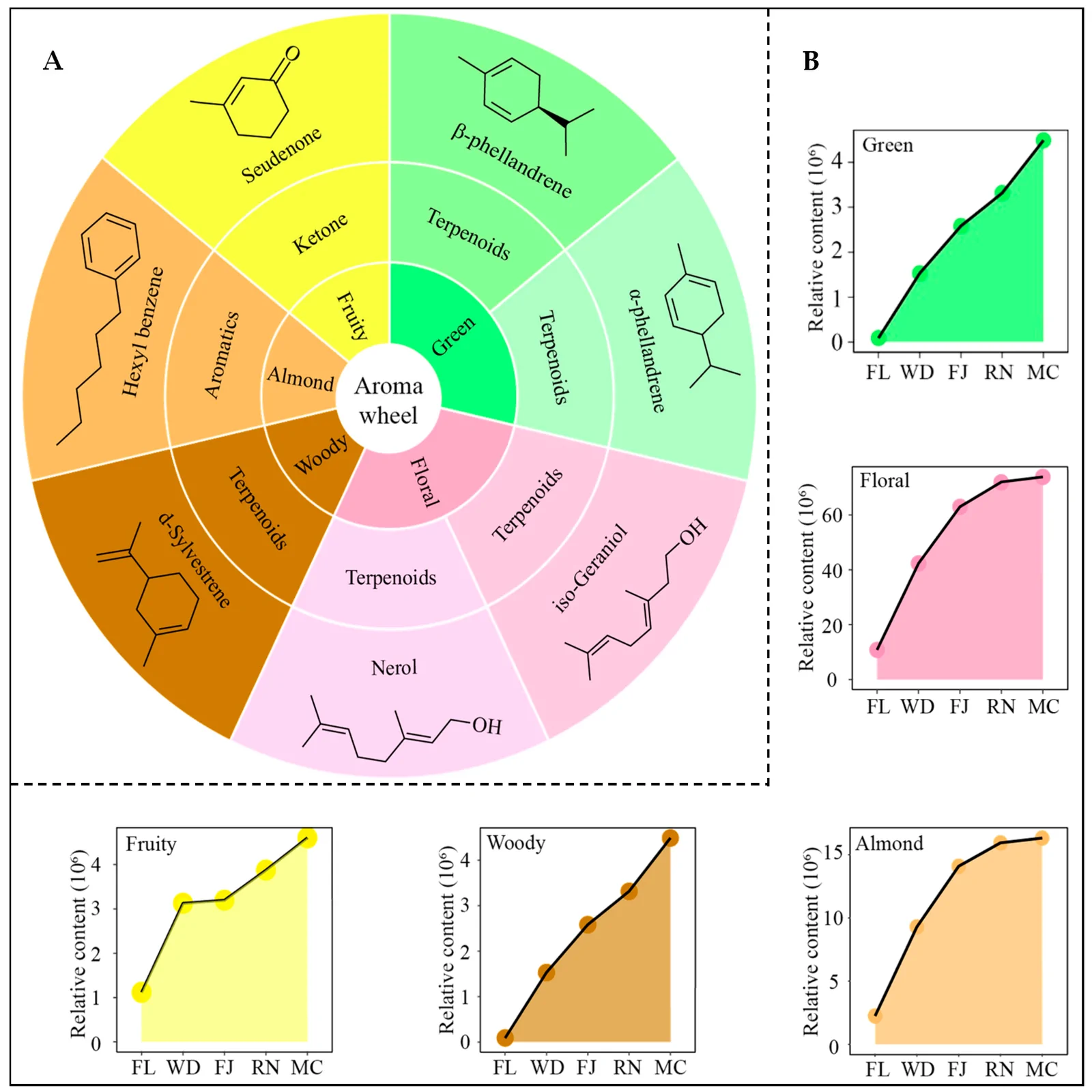
Aroma wheel of space-induced mutant Dahongpao tea and the effect of processing on odor attribute intensity. FL: Fresh leaves; WD: Withering; FJ: Fermentation; RN: Kneading; MC: Drying. (**A**) Aroma wheel of space-induced mutant Dahongpao tea constructed based on the odor characteristics of characteristic VOCs. (**B**) Effects of different processing stages on the intensity of odor attributes in space-induced mutant Dahongpao tea.

Based on the above analyses, an aroma wheel was constructed for the characteristic VOCs of space-induced mutant Dahongpao tea (Figure 4A). These characteristic VOCs mainly exhibited five typical odor attributes, such as green, floral, woody, almond, and fruity. Further analysis revealed (Figure 4B) that as the processing progressed, the green and woody odor attributes continuously intensified, indicating that the processing procedure favored the gradual accumulation of these two aroma characteristics. The floral and almond odor attributes increased rapidly before fermentation and stabilized after fermentation. During tea processing, enzymatic reactions dominate prior to fermentation, whereas after fermentation, the application of heat treatment inactivates enzyme activity, making non-enzymatic reactions the primary transformation pathway [34,35]. Therefore, the formation of floral and almond notes in space-induced mutant Dahongpao tea may be highly dependent on enzymatic reactions. In addition, the fruity attribute increased rapidly before withering, remained stable from withering to fermentation, and intensified significantly again after fermentation. After plucking, withering aims to promote moisture loss, while the rolling and drying steps following fermentation are also accompanied by cell sap exudation and further reduction in moisture content [10]. Thus, the formation of the fruity characteristic in space-induced mutant Dahongpao tea may be closely associated with the gradual reduction in moisture content during processing.

Further analysis of the transformation relationships among the seven characteristic VOCs revealed (Figure S4) that iso-geraniol and nerol, as isomers, jointly contribute to the floral attribute. Based on their structural features and known reaction mechanisms, it is speculated that these two compounds could undergo dehydration and cyclization to yield α-phellandrene and β-phellandrene [36]. The latter two monoterpenes (C_10_H_16_) primarily exhibit green odors, while hexylbenzene, with its almond-like odor, is more likely derived from alternative routes such as fatty acid degradation [37]. In addition, the transformation of β-phellandrene to d-sylvestrene (both C_10_H_16_) represents an isomerization (structural rearrangement). Furthermore, d-sylvestrene could potentially undergo oxidative carbon–carbon cleavage to yield seudenone (C_7_H_10_O) [38], a compound contributing to fruity notes. It should be emphasized that these proposed transformations involve complex biotransformations that are unlikely to occur spontaneously during standard thermal processing (e.g., drying/kneading) without specific enzymatic catalysis, which is largely inactivated after the fermentation stage. Consequently, the inferred conversion network should be regarded as a working hypothesis for future experimental validation. Overall, during the processing of the space-induced mutant Dahongpao tea, iso-geraniol and nerol are suggested as key precursors that gradually accumulate and may undergo further transformations into other characteristic VOCs, synergistically enhancing multiple odor attributes (green, woody, almond, fruity) and collectively shaping the complex aroma profile.

### 3.4. Molecular Docking Simulation of Human Olfactory Receptors with Characteristic VOCs

Binding energy is an important indicator of the spontaneous binding ability between a ligand and a receptor. When the binding energy is below 0 kcal/mol, it indicates that binding can occur; generally, a binding energy lower than −6.0 kcal/mol is considered to represent strong binding affinity [14,39]. Based on this, the present study systematically evaluated the interaction ability and binding modes between the seven characteristic VOCs and 428 human olfactory receptor proteins using molecular docking technology. The results showed (Table S2) that among the 428 human olfactory receptor proteins, 425 were able to bind with all seven characteristic VOCs, with binding energies ranging from −0.23 to −7.80 kcal/mol. The number of olfactory receptors exhibiting binding energies lower than −6.0 kcal/mol with the characteristic VOCs ranged from 13 to 129, among which hexyl benzene (129), α-phellandrene (129), β-phellandrene (127), and d-sylvestrene (126) each exceeded 100, while iso-geraniol (73), nerol (71), and seudenone (13) had relatively fewer. These results indicate that the binding abilities of different characteristic VOCs with human olfactory receptors differ significantly. Combined with the aroma analysis results (Figure 4A), hexyl benzene (almond attribute), α-/β-phellandrene (green attribute), and d-sylvestrene (woody attribute) can form strong-affinity interactions with a large number of olfactory receptors, and may be more easily perceived. In contrast, iso-geraniol and nerol (floral attribute) and seudenone (fruity attribute), especially the latter, bind to fewer strong-affinity receptors. This suggests that in the final product of space-induced mutant Dahongpao tea, the green, woody, and almond odor attributes may be more easily perceived by the human body, whereas the floral and fruity attributes are relatively weaker.

**Figure 5:**
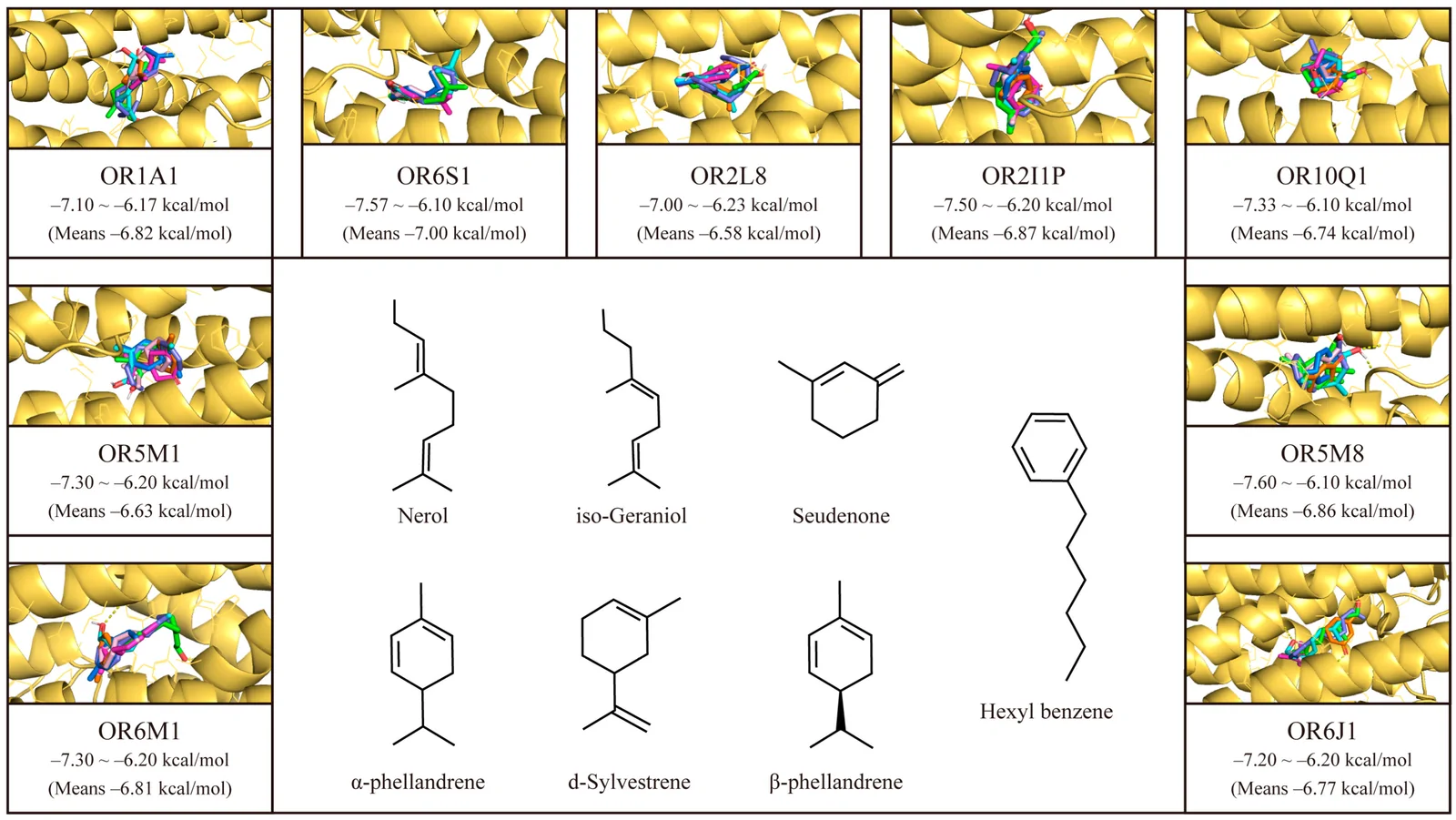
Molecular docking simulation analysis of binding energies between seven characteristic VOCs and nine human olfactory receptor proteins.

Further analysis (Figure 5) revealed that nine human olfactory receptor proteins could form relatively stable, strong binding (<−6.0 kcal/mol) with all seven characteristic VOCs, with average binding energies for each receptor protein ranging from −6.58 to −7.00 kcal/mol, generally close to each other. Among them, OR6S1 exhibited the highest average binding energy (−7.00 kcal/mol), while OR2L8 exhibited the lowest (−6.58 kcal/mol). This indicates that these nine olfactory receptors have broad-spectrum binding ability toward the seven characteristic VOCs and can simultaneously perceive multiple odor attributes. Although the differences in binding strength are subtle, OR6S1 shows the strongest relative binding capacity. Previous studies have shown that olfactory receptors often exhibit cross-recognition ability toward structurally similar ligands [40]. The phenomenon in this study, where multiple characteristic VOCs share the same receptor, is consistent with this finding, suggesting that the complex aroma of space-induced mutant Dahongpao tea may be mediated by the synergistic action of a small number of broad-spectrum receptors with multiple ligands. In summary, molecular docking simulation reveals the binding selectivity between characteristic VOCs and olfactory receptors, providing a theoretical basis for understanding the molecular perception mechanism of the aroma quality of this tea.

### 3.5. Characteristic VOCs, Their Best Predicted Olfactory Receptors, and the Interaction Mechanisms

**Figure 6:**
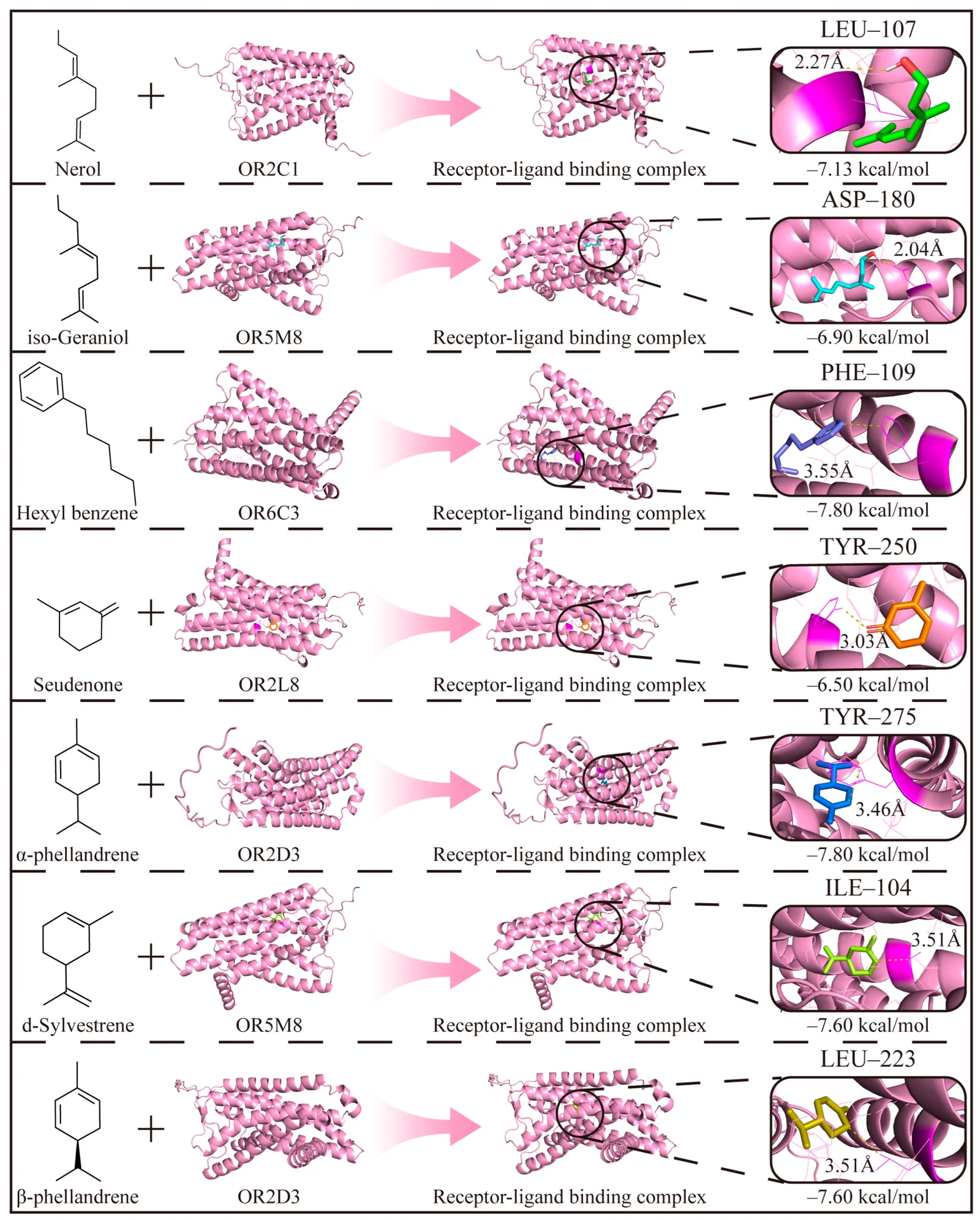
Molecular docking simulation analysis of the interaction mechanisms between seven characteristic VOCs and the best predicted human olfactory receptors.

In olfactory perception, spatial complementarity between ligands and receptors serves as an important basis for molecular recognition [41]. The closest contact distance between a ligand and the receptor binding pocket reflects the degree of spatial conformational matching; a shorter distance generally indicates tighter binding and better spatial complementarity [42]. Based on this, the present study further analyzed the closest contact distances and binding modes between the seven characteristic VOCs and their respective best-fit predicted human olfactory receptor proteins. The results showed (Figure 6, Table S3) that d-sylvestrene and iso-geraniol both exhibited OR5M8 as their best predicted olfactory receptor, with binding sites at ILE-104 and ASP-180, binding energies of −7.60 and −6.90 kcal/mol, and closest contact distances of 3.51 and 2.04 Å, respectively. α-Phellandrene and β-phellandrene both shared OR2D3 as their best predicted receptor, with binding sites at TYR-275 and LEU-223, binding energies of −7.80 and −7.60 kcal/mol, and closest contact distances of 3.46 and 3.51 Å, respectively. The best predicted receptors for hexyl benzene, nerol, and seudenone were OR6C3, OR2C1, and OR2L8, respectively, with binding sites at PHE-109, LEU-107, and TYR-250, binding energies of −7.80, −7.13, and −6.50 kcal/mol, and closest contact distances of 3.55, 2.27, and 3.03 Å, respectively. Further comparison revealed that the four VOCs exhibiting green, woody, and almond odors had binding energies higher than −7.50 kcal/mol (range: −7.60 to −7.80, mean: −7.70 kcal/mol) with their respective best predicted receptors, and a mean closest contact distance of 3.51 Å (range: 3.46–3.55 Å). In contrast, the three VOCs exhibiting floral and fruity odors showed relatively lower binding energies; the average binding energy for floral compounds was −7.02 kcal/mol with a mean closest contact distance of 2.16 Å, while the fruity compound had the lowest binding energy (−6.50 kcal/mol) with a closest contact distance of 3.03 Å.

These results further indicate that during the processing of space-induced mutant Dahongpao tea, VOCs with green, woody, and almond odor characteristics generally exhibit higher binding energies with their best predicted receptors than those with floral and fruity characteristics, suggesting that the former possess stronger receptor affinity and are more readily perceived by the olfactory system. Notably, although the floral and fruity VOCs displayed closer contact distances (especially for floral compounds, with a mean of only 2.16 Å), their binding energies were comparatively weaker. This phenomenon may be attributed to steric hindrance or van der Waals repulsion arising from excessively close proximity, or to the absence of favorable interactions such as hydrogen bonding that could stabilize binding [43,44]. Previous studies have demonstrated that ligand–receptor binding strength depends not only on distance but also on multiple factors, including the chemical properties of residues within the binding pocket, hydrophobic interactions, and electrostatic complementarity [45]. Therefore, although floral and fruity VOCs exhibit high conformational complementarity, their overall lower binding energies place them at a relative disadvantage in perceptual competition.

## 4. Conclusions

**Figure 7:**
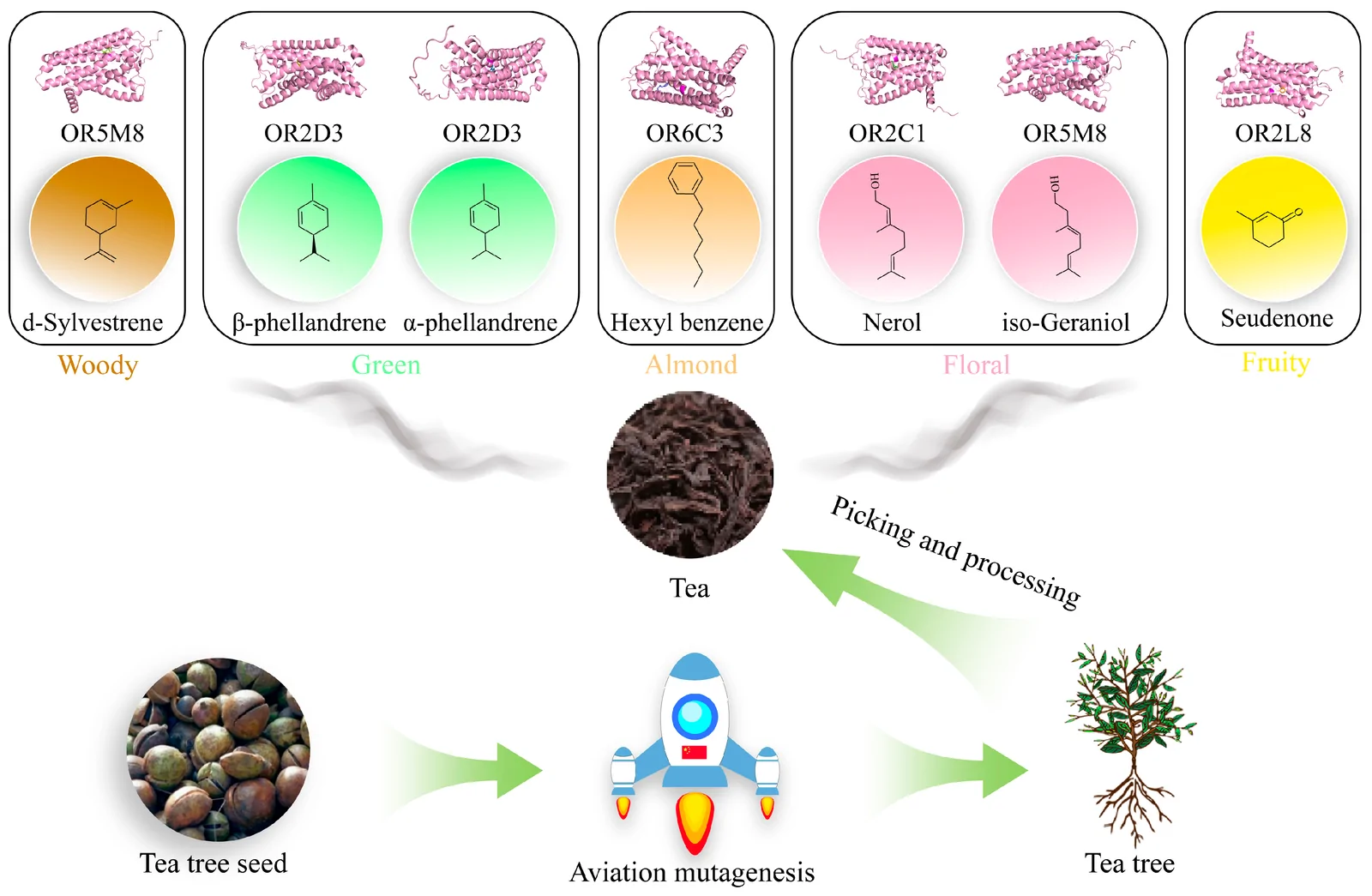
Characteristic VOCs, aroma, and olfactory perception mechanism during the processing of space-induced mutant Dahongpao tea.

This study systematically elucidated the dynamic changes in VOCs, the transformation network of characteristic aroma compounds, and their interaction mechanisms with human olfactory receptors during the processing of space-induced mutant Dahongpao tea (Figure 7). The results showed that the processing procedure significantly reshaped the VOC profile of space-induced mutant Dahongpao. A total of 1103 VOCs were detected, of which 975 exhibited significant differences across processing stages, with the fermentation stage exerting the most pronounced effect. Terpenoids represented the most active aroma pathway during processing. Seven characteristic VOCs (α-phellandrene, β-phellandrene, d-sylvestrene, iso-geraniol, seudenone, hexyl benzene, and nerol) were screened using an OPLS-DA model, contributing green, floral, woody, almond, and fruity odor attributes, respectively. Aroma dynamic analysis revealed that green and woody notes continuously intensified; floral and almond notes increased rapidly before fermentation and then stabilized; and fruity notes increased before withering and intensified again after fermentation. A predicted transformation network was constructed with iso-geraniol and nerol as precursors, as shown, iso-geraniol and nerol undergo dehydration/cyclization to generate α-/β-phellandrene, which are further converted into hexyl benzene, d-sylvestrene, and seudenone, thereby linking the evolution pathway from floral to green to almond/woody to fruity. Molecular docking prediction demonstrated that VOCs exhibiting green, woody, and almond characteristics could form strong binding (binding energy < −6.0 kcal/mol) with more than 100 olfactory receptors, whereas those with floral and fruity characteristics bound strongly to only 13–73 receptors. This indicates that during the processing of space-induced mutant Dahongpao tea, the green, woody, and almond odor attributes may be more easily perceived by the human olfactory system, while the floral and fruity attributes are relatively weaker. In summary, this study clarifies the formation and perception mechanisms of characteristic aroma during the processing of space-induced mutant Dahongpao tea at the molecular level, providing a theoretical basis for the aroma quality evaluation of space-induced mutant tea cultivars. However, the molecular docking results presented in this study can only predict the ligand and receptor binding potential and do not directly correspond to genuine olfactory perception. Specifically, molecular docking calculates binding affinity (thermodynamic potential) but does not model whether a ligand acts as an agonist or an antagonist on the olfactory receptor. Consequently, further validation via sensory evaluation and electrophysiological experiments is still required. Moreover, human olfactory perception also depends significantly on the compound’s volatility and its concentration relative to its odor threshold; therefore, a compound with weaker predicted binding energy but high volatility and abundance may still dominate the overall sensory profile over a tightly binding but low-volatility compound. Furthermore, the VOC transformation network constructed here is primarily inferred from precursor structures and thus represents a hypothetical model that remains to be confirmed by experimental approaches such as isotope tracing or in vitro enzymatic reactions.

## Data Availability

The original contributions presented in this study are included in the article/Supplementary Materials. Further inquiries can be directed to the corresponding authors.

## Supplementary Materials

The following supporting information can be downloaded at: https://www.mdpi.com/article/10.3390/foods15162816/s1, Figure S1. Planting the base of space-induced mutant Dahongpao tea plants. Figure S2. Processing flow chart and processing parameters of space-induced mutant Dahongpao tea. Figure S3. Classification and content analysis of differential VOCs during the processing of space-induced mutant Dahongpao tea. Figure S4. Prediction of transformation relationships among characteristic VOCs. Table S1. Raw VOCs data of space-induced mutant Dahongpao tea at different processing stages. Table S2. Binding energies between seven characteristic VOCs and 428 human olfactory receptors. Table S3. Binding energies between seven characteristic VOCs and the best predicted human olfactory receptors.

## References

[1] Li Y., Luo Q., Qin M., Xu W., Wang X., Zhou J., He C., Chen Y., Yu Z., Ni D. (2024). Study on color, aroma, and taste formation mechanism of large-leaf yellow tea during an innovative manufacturing process. Food Chem..

[2] Xie H., Xiao H., Hu T., Chen G., Liu Y., Ou X., Jiang R., Yu L., Li Q., Huang J. (2024). Effects of different charcoal baking times on the sensory quality and volatile compounds of Dahongpao. J. Tea Sci..

[3] Wu Q., Zhou Z., He J., Zhao S., Ruan S., Liu X., Yu S., Sun Y. (2024). Analysis of aroma precursors in Jinmudan fresh tea leaves and dynamic change of fatty acid volatiles during black tea processing. Food Chem. X.

[4] Wang J., Li Z. (2024). Effects of processing technology on tea quality analyzed using high-resolution mass spectrometry-based metabolomics. Food Chem..

[5] Ali S., Suryakant T.N. (2024). Mutation breeding and its importance in modern plant breeding: A review. J. Exp. Agric. Int..

[6] Kashtwari M., Mansoor S., Wani A.A., Najar M.A., Deshmukh R.K., Baloch F.S., Abidi I., Zargar S.M. (2022). Random mutagenesis in vegetatively propagated crops: Opportunities, challenges and genome editing prospects. Mol. Biol. Rep..

[7] Yali W., Mitiku T. (2022). Mutation breeding and its importance in modern plant breeding. J. Plant Sci..

[8] Ye J., Zhang Q., Cheng P., Wang Y., Zou J., Lin S., Li M., Jia M., Chen Y., Jia X. (2024). Aviation mutagenesis alters the content of volatile compounds in Dahongpao (*Camellia sinensis*) leaves and improves tea quality. Foods.

[9] Jia X., Wang Y., Li Q., Zhang Q., Zhang Y., Lin S., Cheng P., Chen M., Du M., Ye J. (2023). Contribution of traditional deep fermentation to volatile metabolites and odor characteristics of Wuyi rock tea. Front. Bioeng. Biotech..

[10] Qi D., Shi Y., Lu M., Ma C., Dong C. (2024). Effect of withering/spreading on the physical and chemical properties of tea: A review. Compr. Rev. Food Sci. Food Saf..

[11] Zeng Q., Wang H., Tuo J., Ding Y., Cao H., Yue C. (2025). Volatile organic compounds in teas: Identification, extraction, analysis, and application of tea aroma. Foods.

[12] Liang Y., Wang Z., Zhang L., Dai H., Wu W., Zheng Z., Lin F., Xu J., Huang Y., Sun W. (2024). Characterization of volatile compounds and identification of key aroma compounds in different aroma types of Rougui Wuyi rock tea. Food Chem..

[13] Wang J., Zhang Q., Fan W., Shi Q., Mao J., Xie J., Chai G., Zhang C., Zhang C. (2025). Deciphering olfactory receptor binding mechanisms: A structural and dynamic perspective on olfactory receptors. Front. Mol. Biosci..

[14] Zhou X., Ni J., Ge W., Wang X., Li Y., Wang H., Ma C. (2024). Machine learning-based virtual screening of multi-target anti-obesity compounds from medicinal and edible plants: A combined in silico and in vitro study. Food Biosci..

[15] Xiao Z., Li Q., Niu Y., She Y., Sun Z., Zhang J., Wang Z., Zhou R., Zhu J. (2024). Mechanism of the interaction between olfactory receptors and characteristic aroma compounds in sweet orange juice. LWT.

[16] Zeng S., Zhang L., Li P., Pu D., Fu Y., Zheng R., Xi H., Qiao K., Wang D., Sun B. (2023). Molecular mechanisms of caramel-like odorant-olfactory receptor interactions based on a computational chemistry approach. Food Res. Int..

[17] Ye J., Wang H. (2025). Wuyi Rock Tea Germplasm Resources, Processing, and Evaluation.

[18] Yuan H., Cao G., Hou X., Huang M., Du P., Tan T., Zhang Y., Zhou H., Liu X., Liu L. (2022). Development of a widely targeted volatilomics method for profiling volatilomes in plants. Mol. Plant.

[19] Wang Y., Sun Y., Joseph P.V. (2023). Diverse evolutionary rates and gene duplication patterns among families of functional olfactory receptor genes in humans. PLoS ONE.

[20] Le Guilloux V., Schmidtke P., Tuffery P. (2009). Fpocket: An open source platform for ligand pocket detection. BMC Bioinform..

[21] Trott O., Olson A.J. (2010). AutoDock Vina: Improving the speed and accuracy of docking with a new scoring function, efficient optimization, and multithreading. J. Comput. Chem..

[22] Wickham H. (2019). Advanced R.

[23] Ye J., Wang Y., Lin S., Hong L., Kang J., Chen Y., Li M., Jia Y., Jia X., Wu Z. (2023). Effect of processing on aroma intensity and odor characteristic of Shuixian (*Camellia sinensis*) tea. Food Chem. X.

[24] Yang Y., Xie J., Wang Q., Deng Y., Zhu L., Zhu J., Yuan H., Jiang Y. (2024). Understanding the dynamic changes of volatile and non-volatile metabolites in black tea during processing by integrated volatolomics and UHPLC-HRMS analysis. Food Chem..

[25] Wong M., Sirisena S., Ng K. (2022). Phytochemical profile of differently processed tea: A review. J. Food Sci..

[26] Liu L., Shi J., Yuan Y., Yue T. (2022). Changes in the metabolite composition and enzyme activity of fermented tea during processing. Food Res. Int..

[27] Liu Y., Chen Q., Liu D., Yang L., Hu W., Kuang L., Huang Y., Teng J., Liu Y. (2023). Multi-omics and enzyme activity analysis of flavour substances formation: Major metabolic pathways alteration during Congou black tea processing. Food Chem..

[28] Wei J., Yang Y., Peng Y., Wang S., Zhang J., Liu X., Liu J., Wen B., Li M. (2023). Biosynthesis and the transcriptional regulation of terpenoids in tea plants (*Camellia sinensis*). Int. J. Mol. Sci..

[29] Yang Y., Deng Q., Jia X., Shi J., Wan C., Zhou Q., Wang Q. (2021). Characterization of key odorants in peeled and unpeeled flaxseed powders using solvent-assisted flavor evaporation and odor activity value calculation. LWT.

[30] Dziadas M., Jeleń H.H. (2016). Comparison of enzymatic and acid hydrolysis of bound flavor compounds in model system and grapes. Food Chem..

[31] Gao Y., Lei Z., Huang J., Sun Y., Liu S., Yao L., Liu J., Liu W., Liu Y., Chen Y. (2024). Characterization of key odorants in Lushan Yunwu tea in response to intercropping with flowering cherry. Foods.

[32] Valdés García A., Sánchez Romero R., Juan Polo A., Prats Moya S., Maestre Pérez S.E., Beltrán Sanahuja A. (2021). Volatile profile of nuts, key odorants and analytical methods for quantification. Foods.

[33] Artik N., Akan S., Okay Y., Durmaz N., Köksal A.İ. (2021). Volatile aroma component of natural and roasted hazelnut varieties using solid-phase microextraction gas chromatography/mass spectrometry. Acta Sci. Pol. Hortorum Cultus.

[34] Hoque A., Padhiary M., Prasad G., Tiwari A. (2024). Optimization of fermentation time, temperature, and tea bed thickness in CFM to enhance the biological composition of CTC black tea. J. Inst. Eng. India Ser. A.

[35] Zhang Y., Yan K., Peng Q., Feng S., Zhao Z., Chen L., Ye L., Fu J., Lv H., Mu D. (2024). Insights into major pigment accumulation and (non) enzymatic degradations and conjugations to characterized flavors during intelligent black tea processing. Food Chem..

[36] Eisenacher M., Beschnitt S., Hölderich W. (2012). Novel route to a fruitful mixture of terpene fragrances in particular phellandrene starting from natural feedstock geraniol using weak acidic boron based catalyst. Catal. Commun..

[37] Stefanovic E., Kilcawley K.N., Rea M.C., Fitzgerald G.F., McAuliffe O. (2017). Genetic, enzymatic and metabolite profiling of the *Lactobacillus casei* group reveals strain biodiversity and potential applications for flavour diversification. J. Appl. Microbiol..

[38] Silvestre A.J., Válega M., Cavaleiro J.A. (2000). Chemical transformation of 1, 8-cineole: Synthesis of seudenone, an insect pheromone. Ind. Crop. Prod..

[39] Mei S., Ding J., Chen X. (2023). Identification of differential volatile and non-volatile compounds in coffee leaves prepared from different tea processing steps using HS-SPME/GC–MS and HPLC-Orbitrap-MS/MS and investigation of the binding mechanism of key phytochemicals with olfactory and taste receptors using molecular docking. Food Res. Int..

[40] Liu Y.N., Gao J.J., Zhuang X.L., Wu D.D., Sun Y.B. (2026). Near complete assembly of *Drosophila melanogaster* Canton S strain genome. Nat. Commun..

[41] Zhang L., Wu Y., Gong Z., Yuan J., Wang W., Xia Y., Yang Y., Lin Y., Luo Z., Cao X. (2026). Receptor-mimetic stereo olfaction for simultaneous odor recognition and spatial localization. Adv. Mater..

[42] Pallante L., Cannariato M., Androutsos L., Zizzi E.A., Bompotas A., Hada X., Grasso G., Kalogeras A., Mavroudi S., Benedetto G.D. (2024). VirtuousPocketome: A computational tool for screening protein–ligand complexes to identify similar binding sites. Sci. Rep..

[43] Schmiedeberg K., Shirokova E., Weber H.P., Schilling B., Meyerhof W., Krautwurst D. (2007). Structural determinants of odorant recognition by the human olfactory receptors OR1A1 and OR1A2. J. Struct. Biol..

[44] Sharma A., Saha B.K., Kumar R., Varadwaj P.K. (2022). OlfactionBase: A repository to explore odors, odorants, olfactory receptors and odorant–receptor interactions. Nucleic Acids Res..

[45] Block E. (2018). Molecular basis of mammalian odor discrimination: A status report. J. Agric. Food Chem..

