# Supplementary material for: Evolution of Characteristic Aroma During Processing of Space-Induced Mutant Dahongpao Tea and the Preliminary Exploration of Its Olfactory Perception
Source: Foods. 2026 Aug 12;15(16):2816. doi: 10.3390/foods15162816 (PMC13512566; doi:10.3390/foods15162816)
Supplement: Supplementary file 1 [file foods-15-02816-s001.zip › Supplementary data (Figures S1~S4).pdf]

Supplementary data

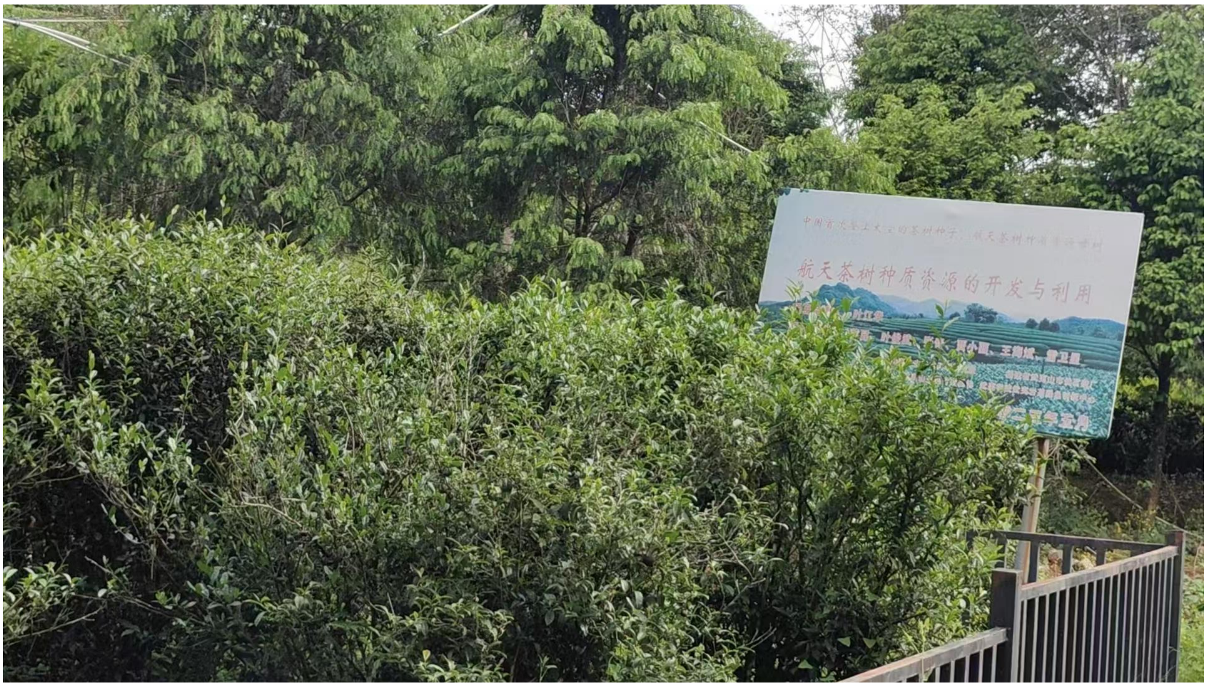

Figure S1. Planting base of space-induced mutant Dahongpao tea plants

Note: The process of aerospace mutagenesis and cultivation of Dahongpao tea tree seeds was that at 5:58 on November 1, 2011, Dahongpao tea tree seeds were launched with the unmanned spacecraft "Shenzhou VIII", and two days later, "Shenzhou VIII" was in space rendezvous and docking with the target vehicle "Tiangong I". After 12 days of combined body operation, at 18:30 on November 16, 2011, the Shenzhou VIII spacecraft and the Tiangong 1 target vehicle were successfully separated, and the return capsule returned to the ground at 19:32 on November 17, 2011, with a total flight time of 16 days, 13 hours and 34 minutes. In April 2012, the tea tree seeds after aerospace mutagenesis were planted in the soil.

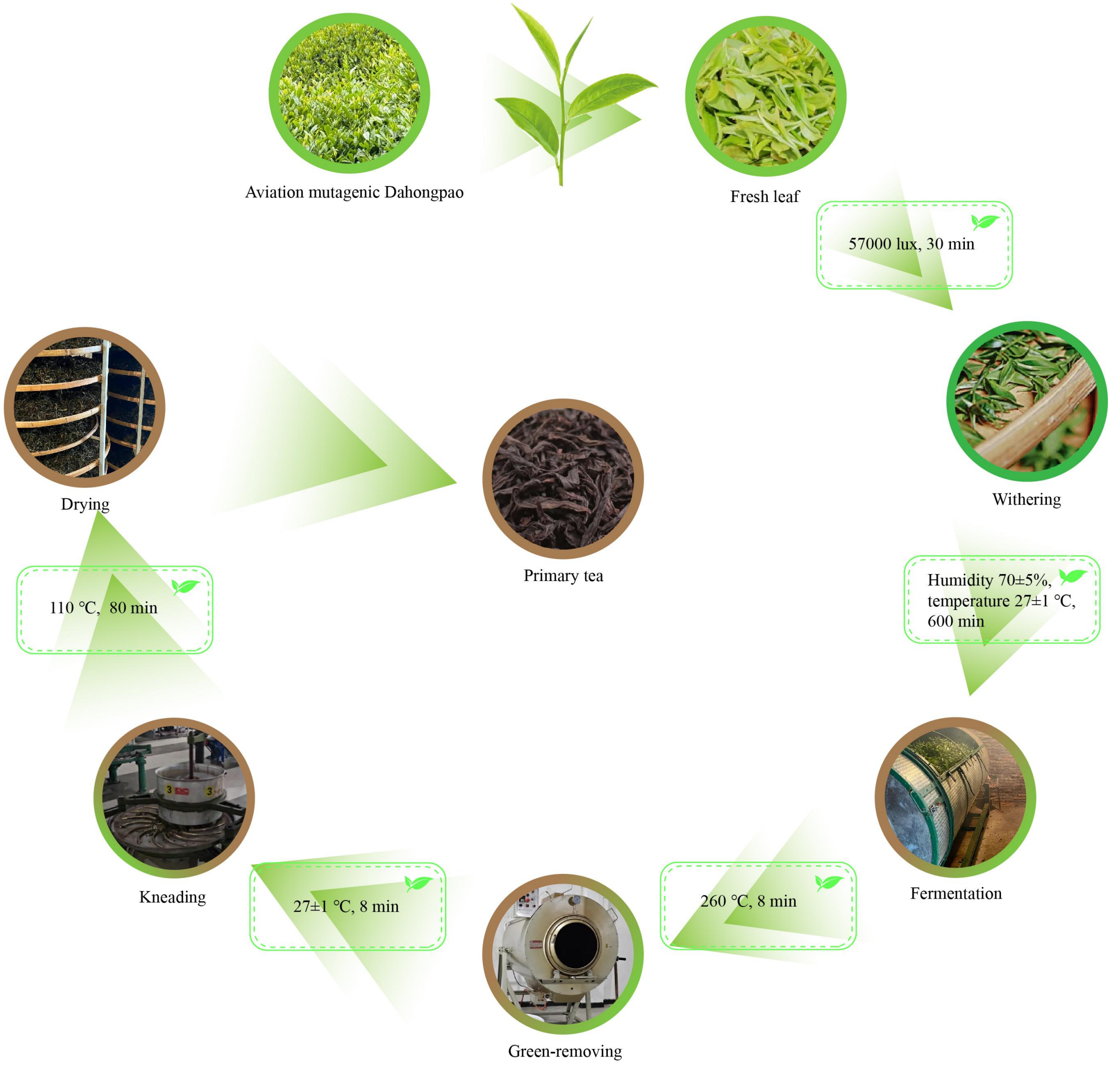

Figure S2. Processing flow chart and processing parameters of space-induced mutant Dahongpao tea

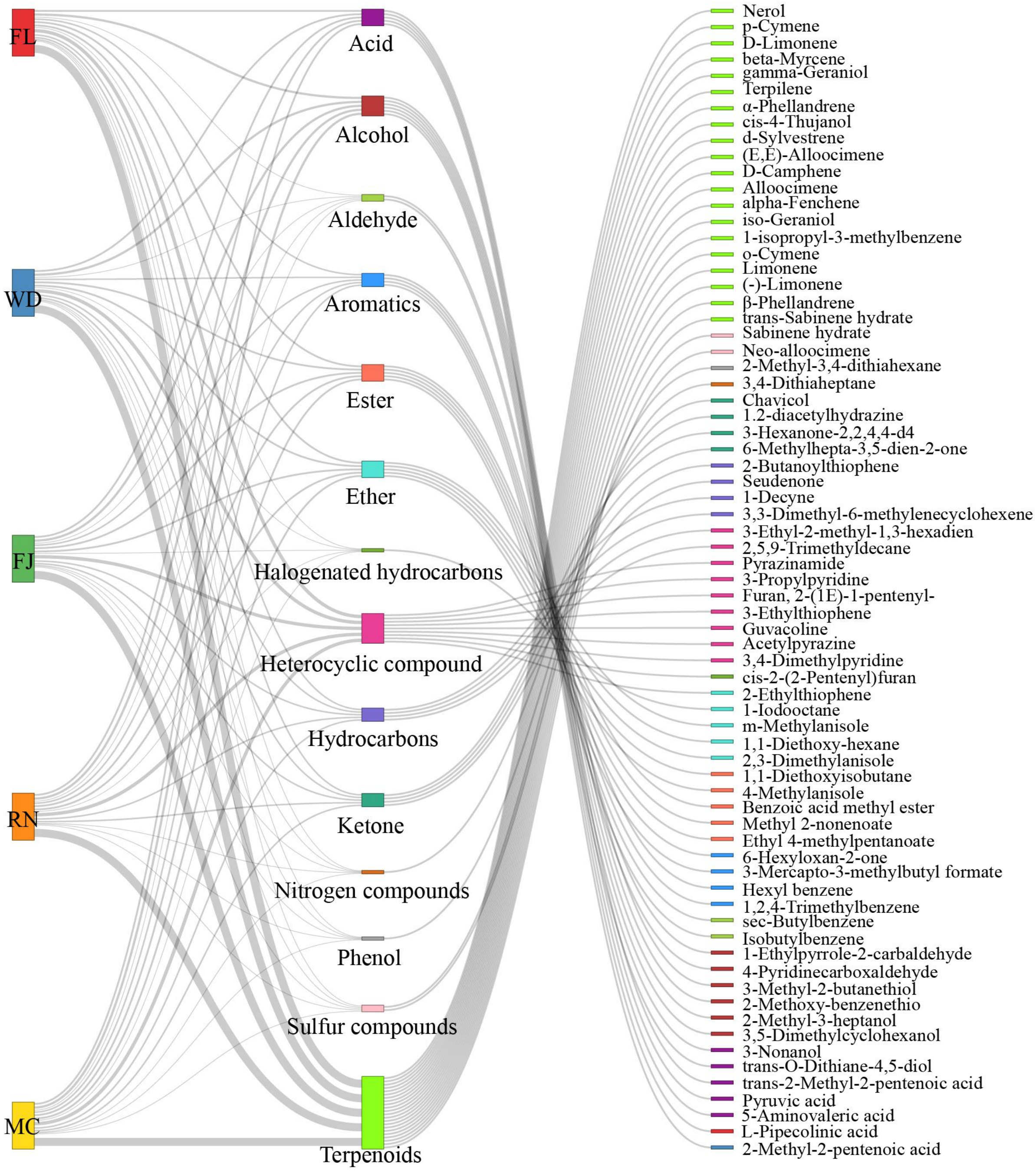

Figure S3. Classification and content analysis of differential VOCs during the processing of space-induced mutant Dahongpao tea

Note: FL: Fresh leaves; WD: Withering; FJ: Fermentation; RN: Kneading; MC: Drying.

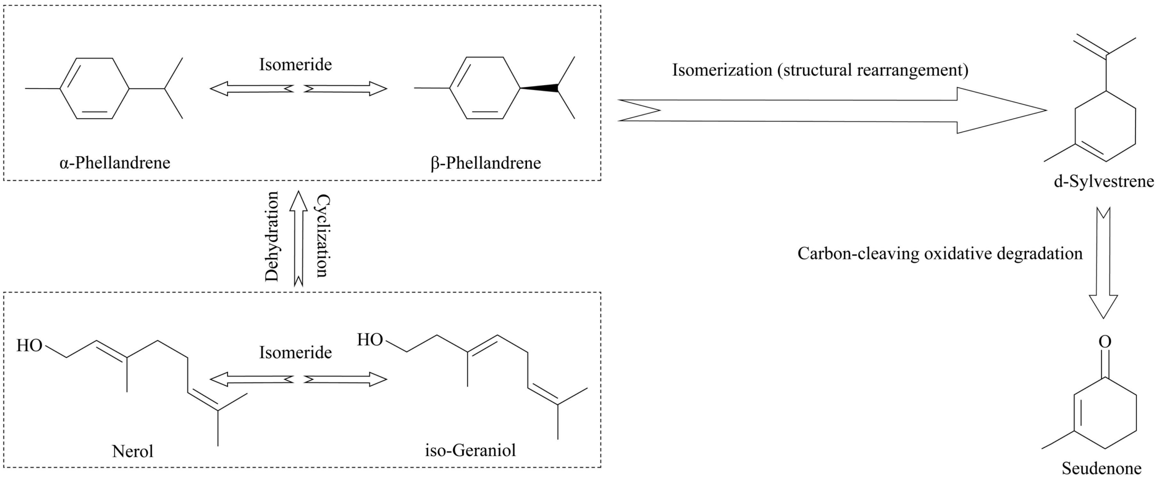

Figure S4. Prediction of transformation relationships among characteristic VOCs
